# The Predictive Value of 8-Hydroxy-Deoxyguanosine (8-OHdG) Serum Concentrations in Irradiated Non-Small Cell Lung Carcinoma (NSCLC) Patients

**DOI:** 10.3390/biomedicines12010134

**Published:** 2024-01-09

**Authors:** Kyriakos Orfanakos, Constantinos E. Alifieris, Emmanouil K. Verigos, Maria V. Deligiorgi, Kosmas E. Verigos, Mihalis I. Panayiotidis, Michail Nikolaou, Dimitrios T. Trafalis

**Affiliations:** 1 Laboratory of Pharmacology, Faculty of Medicine, National and Kapodistrian University of Athens, 11527 Athens, Greece; kyriakosorfanakos@gmail.com (K.O.); mdeligiorgi@yahoo.com (M.V.D.); kosmas.verigos@usa.net (K.E.V.); dtrafal@med.uoa.gr (D.T.T.); 2 Department of Radiation Therapy, 401 General Military Hospital, 11525 Athens, Greece; 3 Department of Hepatobiliary and Transplant Surgery, St. Vincent’s University Hospital, D04 T6F4 Dublin, Ireland; 4 Department of Radiation Oncology, General Anticancer Oncology Hospital of Athens “O Agios Savvas”, 11522 Athens, Greece; 5 Department of Cancer Genetics, Therapeutics & Ultrastructural Pathology, The Cyprus Institute of Neurology & Genetics, Nicosia 2371, Cyprus

**Keywords:** ionizing radiation, biomarker, 8-OHdG, 8-hydroxy-2-deoxyguanosine, lung cancer

## Abstract

Ionizing radiation is strongly linked to direct or indirect DNA damage, as with the production of reactive oxygen species (ROS), which in turn produce DNA damage products, such as 8-hydroxy-2-deoxyguanosine (8-OHdG). In this study, we aimed to investigate the formation of 8-OHdG after irradiation in patients with non-small cell cancer (NSCLC) and its use as a biomarker. Sixteen patients with squamous and thirty-six patients with non-squamous pathology were included. An enzyme-linked-immunosorbent assay (ELISA) was performed before and after radiation. A dose-dependent relationship was confirmed: 8-OHdG plasma concentrations, increased in the total of NSCLC patients and specifically with a linear correlation in non-squamous pathology; in squamous histology, after an initial increase, a significant decrease followed after 20 Gy dose of irradiation. The pretreatment total irradiated tumor volume (cm^3^) was positively correlated with 8-OHdG levels in patients with squamous histology. When plotting the 8-OHdG plasma concentration at a 10 Gy irradiation dose to the baseline, the AUC was 0.873 (95% CI 0.614–0.984), *p* < 0.0001, with an associated criterion value of >1378 as a cutoff (sensitivity 72.7%, specificity 100%). When normalizing this ratio to BSA, the associated criterion cutoff value was >708 (sensitivity of 100%, specificity 80%). Lastly, 8-OHdG levels were closely related with the development of radiation-induced toxicities.

## 1. Introduction

Lung cancer incidence has declined steadily since 2006–2007 by 2.6% annually in men and 1.1% annually in women, mostly due to differences in smoking cessation [1]. However, still, 238,340 new cancer cases were estimated for 2023 and a 12.2% increase is expected all new cancer diagnoses. Of all cancer cases, lung cancer remains the most lethal cancer for both women and men, with an estimated 127,070 deaths in 2023 (20.8% of all cancer deaths), and the third most common cancer overall after breast and prostate cancer, respectively [2,3]. Lung cancer is largely differentiated histologically in small-cell lung cancer (SCLC) and non-small cell cancer (NSCLC, further divided to squamous and non-squamous pathology) [4].

Ionizing radiation is still widely applied as part of the treatment for many malignant tumors including lung cancer. It is an integral part of treatment as it reduces the risk of local recurrence, increases survival and is often palliative to many symptoms such as pain, obstruction and bleeding. In this way, it can shrink a tumor and destroy cells, making it useful in the adjuvant or neoadjuvant setting.

Radiotherapy is the form of treatment used in cancer patients involving ionizing radiation and most commonly in the form of X- and gamma radiation [5]. Usually, radiation is delivered internally or externally. With external beam-irradiation (EBRT), a linear accelerator produces X-rays, whereas internal irradiation (brachytherapy) is delivered mainly by gamma-radiation sources, like radioactive isotopes that are inserted in the patient’s body. The EBRT dose is usually given in fractions due to increased electrical potential (4–20 mV) and thus concomitant injury to surrounding normal tissue, whereas the internal irradiation produces less potent electrical potential (0.6–1 mV), thus producing a more focal effect, limiting normal tissue damage [6,7,8].

Radiotherapy has direct or indirect effects on all living cells. Specifically, it can directly disrupt the structure of atoms or, indirectly, the production of reactive chemical species that in turn damage DNA, proteins and the lipids of cells. Thus, atoms and molecules that are attacked by radiation release electrons and produce ions which form reactive oxygen and nitrogen species [9]. Water is a major source of reactive oxygen species (ROS) such as superoxide anions (O_2_^•−^), hydroxyl radicals (HO^•^) and hydrogen peroxide (H_2_O_2_) [10]. Ionizing radiation also causes oxidative DNA damage (DNA breaks, base damage and sugar damage) and immunosuppression [11].

The most common base lesion which is induced by ROS is guanine and a hydroxyl group is added to the eighth position of the purine base, leading to the formation of 8-hydroxy-deoxyguanosine (8-OHdG). Due to its significant mutagenic activity, 8-OhdG is the most studied oxidative DNA lesion. To this end, this predominant form of the free radical lesion of DNA is frequently measured as an indication of the extent of DNA damage and oxidative stress [12] and is frequently measured by urinary excretion [13]. ROS can be continuously found for weeks to months in irradiated tissue and cells leading to latent/late injury [14].

The question remains whether 8-OHdG is a promising biomarker of oxidative DNA damage and/or prognostic significance following radiotherapy. In this study, we aim to investigate the formation of 8-OHdG, its measurement in blood samples and its possible correlation to radiation dose in patients with non-small cell lung cancer (NSCLC).

## 2. Materials and Methods

### 2.1. Eligibility Criteria

In this study, inclusion criteria demanded patients older than 18 years old who had (1) cytologically or histologically and radiographically confirmed NSCLC at diagnosis, (2) a disease status measurable by Response Evaluation Criteria in Solid Tumors (RECIST) version 1.1, (3) disease stage IIIA-IIIC as reported by TNM criteria 8th edition [15], (4) Eastern Cooperative Oncology Group (ECOG) PS of 0–2, (5) stage III with inoperable (or contraindicated for surgical therapy) lung cancer patients with indications for radiotherapy. Laboratory values were expected to be within the limits to permit patient to receive radiotherapy. Patients were excluded from the study if they were unable to comply with treatment protocol, they had concurrent malignancies other than NSCLC or if contraindications existed for use of radiation. Previous history of irradiation or work in medical ionizing radiation posts or other types with high risk for ionizing-radiation exposure was also considered an exclusion criterion.

### 2.2. Treatment Schedule

Chemotherapy schedule: Chemotherapy regimen included combination of docetaxel and carboplatin. Chemotherapy was allowed before and/or after radiation but not concurrently to radiation treatment. Docetaxel 75 mg/m^2^ and carboplatin AUC 6 were given every 3 weeks.

Radiotherapy schedule: All patients were firstly subjected to computed tomography scan (CT) and thereby 3D conformal radiation planning was performed in order to provide Intensity-Modulated Radiotherapy (IMRT) delivery technique with usage of up to 6-MV photons. The radiation treatment course followed was a 2 Gy daily fraction dose for a total of 30 fractions. Total dose administered to the tumor and the involved lymph node groups was 60 Gy.

### 2.3. Patient Assessment

All patients at baseline prior to enrollment were examined and a careful history was taken. Performance status was assessed and laboratory tests for organ function were performed. Furthermore, imaging with chest X-ray, CT scan of chest and abdomen or other imaging studies necessary were included. Patients were re-examined at intervals after and during radiotherapy and imaging reobtained as indicated or per protocol. Additionally, clinical patients’ characteristics such as, body surface area (BSA), body mass index (BMI), glomerular filtration rate (GFR), and irradiated tumor volume (TV) were recorded.

Tumor response criteria. The tumor response evaluation was performed according to the criteria of RECIST guideline in Solid Tumors (version 1.1) [16], where complete response (CR) corresponds to the disappearance of all target lesions and reduction in the short axis measurement of all pathologic lymph nodes to ≤10 mm, partial response (PR) corresponds to ≥30% decrease in the sum of the longest diameter of the target lesions compared with baseline, progression of disease (PD) corresponds to ≥20% increase in the sum of the longest diameter of the target lesions compared with the smallest sum of the longest diameter recorded and stable disease (SD) corresponds to neither PR nor PD.

Radiotherapy-induced toxicity assessment. Radiotherapy-induced common toxicities were screened by clinical examination and laboratory tests (e.g., anemia, leukopenia, thrombocytopenia, hyperuricemia, esophagitis, pneumonitis, dyspnea, fatigue, nausea, dermatitis-burn, dysphagia, dehydration, etc.) and classified according to National cancer Institute Common Terminology Criteria for Adverse Effects (NCI-CTCAE version 4.0) [17].

### 2.4. Sample Collection and Preparation

DNA damage products were measured in biological fluids (blood serum) at specific intervals on Day 0, Day 5, Day 8, Day 15 and Day 30 of irradiation treatment schedule, using standard ELISA methods (standard ELISA kits) which detect 8-hydroxy-2′-deoxyguanosine (8-OHdG) levels. Blood samples were obtained with single-use syringes and needles. Afterwards, the specimens were transferred into vacuum tubes without any anticoagulant. Centrifugation was performed at 3000× *g* for 10 min within 10–15 min after blood collection. The precipitates were removed and the supernatant serum was transferred to 1.5 mL labeled centrifuge tubes and stored at −80 °C until analysis.

Blood serum 8-OHdG was quantified using the OxiSelect™ Oxidative DNA Damage ELISA Kit (8-OHdG Quantitation) and Trial Size (CELL BIOLABS, Inc., San Diego, CA, USA). This is a modified competitive ELISA with detection sensitivity ranging from 100 pg/mL to 20 ng/mL. Specimens were handled and processed according to manufacturer’s recommendations. The patient samples were loaded onto an 8-OHdG-BSA conjugate preabsorbed 96-well plate at the same time as the 8-OHdG standards for comparison. Briefly, to coat the 96-well plate with 8-OHdG/BSA conjugate, 1 mg/mL of OHdG conjugate was diluted to 1 μg/mL in 1x PBS solution. Then, 100 µL of the 1 μg/mL of 8-OHdG conjugate were added to each well and the plate was incubated overnight at 4 °C. After the incubation time, the 8-OHdG coating solution was discarded and the plate was washed once with distilled water. After this was completed, 200 µL of assay diluent were added to each well. Following that, the 8-OHdG conjugate was blocked for 1 h at room temperature. The plate was transferred to a temperature of 4 °C and the assay diluent was removed immediately before use. Serial dilutions of 8-OHdG standard were carried out covering a concentration range of 0 ng/mL to 20 ng/mL. The serial dilutions were performed in order to prepare the 8-OHdG standard curve. Afterwards, 50 µL of the tested sample or 8-OHdG standard were added to the corresponding wells of the 8-OHdG conjugate-coated plate and samples were incubated at room temperature for 10 min. a total of 50 µL of the diluted anti-8-OHdG antibody was then added and samples were incubated at room temperature for one hour. After the incubation time had passed, all wells were washed three times with 250 µL of 1× wash buffer, and thereafter, 100 μL/well of the diluted secondary antibody–enzyme conjugate were added. All samples were again incubated at room temperature for 1 h and subsequently all wells were washed 3 times as previously described. The addition of 100 µL of substrate solution was followed by incubation at room temperature from 2 to 30 min. The enzymatic reaction was terminated by the addition of 100 µL of Stop Solution. The absorbance was immediately measured on an ELISA reader at 450 nm (Versamax, Orleans, LA, USA). The experiment was carried out in triplicates to ensure correct measurements and assess variability.

### 2.5. Statistical Analysis

Response evidence was classified as complete response (CR), partial response (PR), stable disease (SD) and progressive disease (PD). In order to evaluate for biomarker efficiency, ROC curve analysis was performed for each of the histologies, squamous or non-squamous. Considering a power of 0.80 and a type 1 error of 0.05, we considered an area under curve of 0.90 to be significantly different from the null hypothesis of 0.5 for twice as many responders than non-responders. For that, we needed at least 14 patients for each histology category.

The patient accrual was consecutive and the analysis was of retrospective fashion. The statistical analysis and processing of the results were performed using Microsoft Excel (Microsoft Hellas, Athens, Greece) and OriginPro (OriginLab Corp., Northampton, MA, USA). The production of 8-OHdG as well as the change in its concentration over time and the doses of radiotherapy were estimated from the logarithmic distribution. The correlation of 8-OHdG with the clinical characteristics of the patients (BMI, age, BSA, GFR and Hct) was evaluated with the linear correlation coefficient r of the Pearson test.

In addition, the correlation of the percent changes of 8-OHdG with the degree of toxicity was examined by linear adjustment. A dose response model as well as a polynomial adaptation was applied to correlate the percent changes 8-OHdG with the correlation coefficient r for the development of radial dermatitis.

The study was performed in accordance with the Europe Convention on human rights and biomedicine (law 2919/1998) and approved by the Hospital’s Scientific Board and the Board of Bioethics of National and Kapodistrian University of Athens (NKUA).

## 3. Results

### 3.1. Patients

Fifty-two patients were consecutively enrolled (37 males and 15 females); these patients with NSCLC at stages IIIA to IIIC underwent loco-regional radiotherapy on primary tumor sites. The age of the recruited patients ranged from 53 to 76 years, with an average age of 66.8 years. A total of 31% of patients were diagnosed with squamous and 69% with non-squamous histology. All of the patients were smokers. In total, 86.5% had received combination chemotherapy (Table 1). All patients were treated and the blood samples were collected in the Department of Radiation Therapy, 401 General Military Hospital, Athens, Greece.

The overall response rate was 68.8% in squamous and 58.3% in non-squamous pathology.

### 3.2. Plasma 8-OHdG Levels in Patients Receiving Radiotherapy

**Figure 1:**
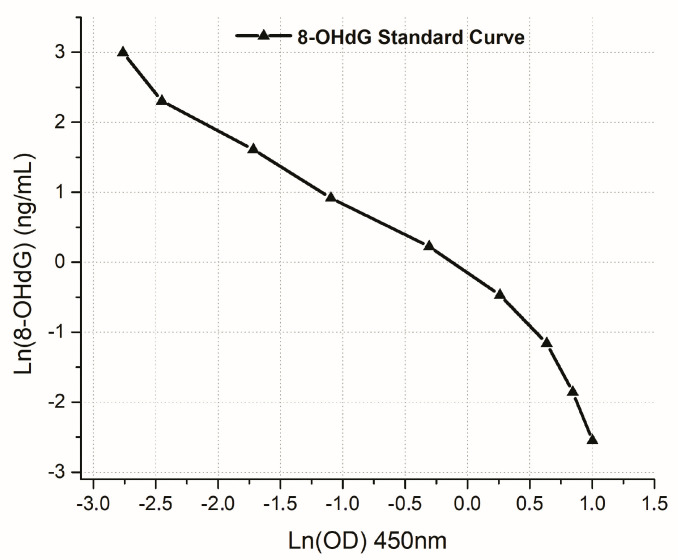
8-OHdG standard curve fitted by logarithmic model. The standard curve was made by plotting the 8-OHdG concentration (ng/mL) against OD (450 nm) values.

**Figure 2:**
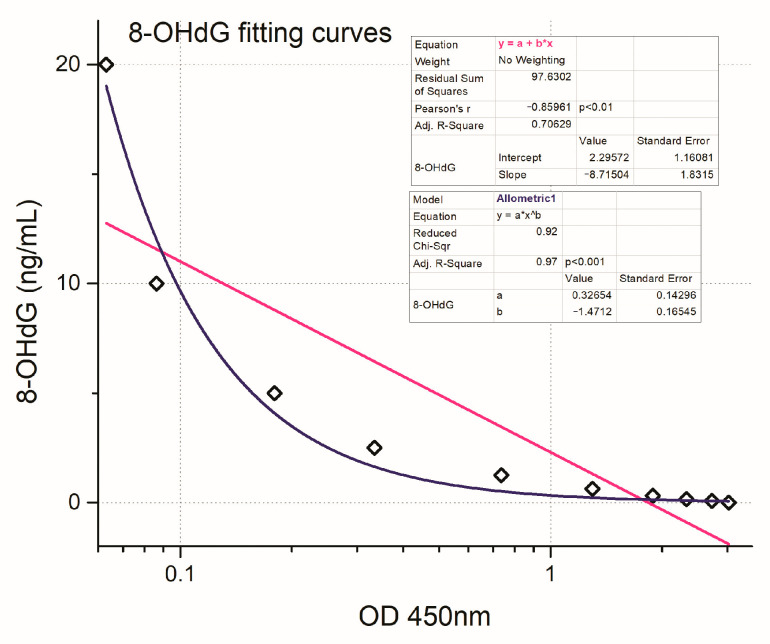
The two 8-OHdG fitting curves were performed using the equations of the linear (y = a + b∗x, Pearson’s r = −0.85961 (*p* < 0.01) and adjusted R-square = 0.70629) and allometric (y = a∗x^b^ with adjusted R-square = 0.97773 (*p* < 0.001) models).

The 8-OHdG concentration (ng/mL) in the plasma samples of patients who received radiotherapy was quantitated according to the 8-OHdG standard curve (Figure 1). Furthermore, according to Figure 2, the two 8-OHdG fitting curves were performed using the equations of the linear and allometric models.

### 3.3. The Pattern of 8-OHdG Levels in Squamous versus Non-Squamous NSCLC

**Figure 3:**
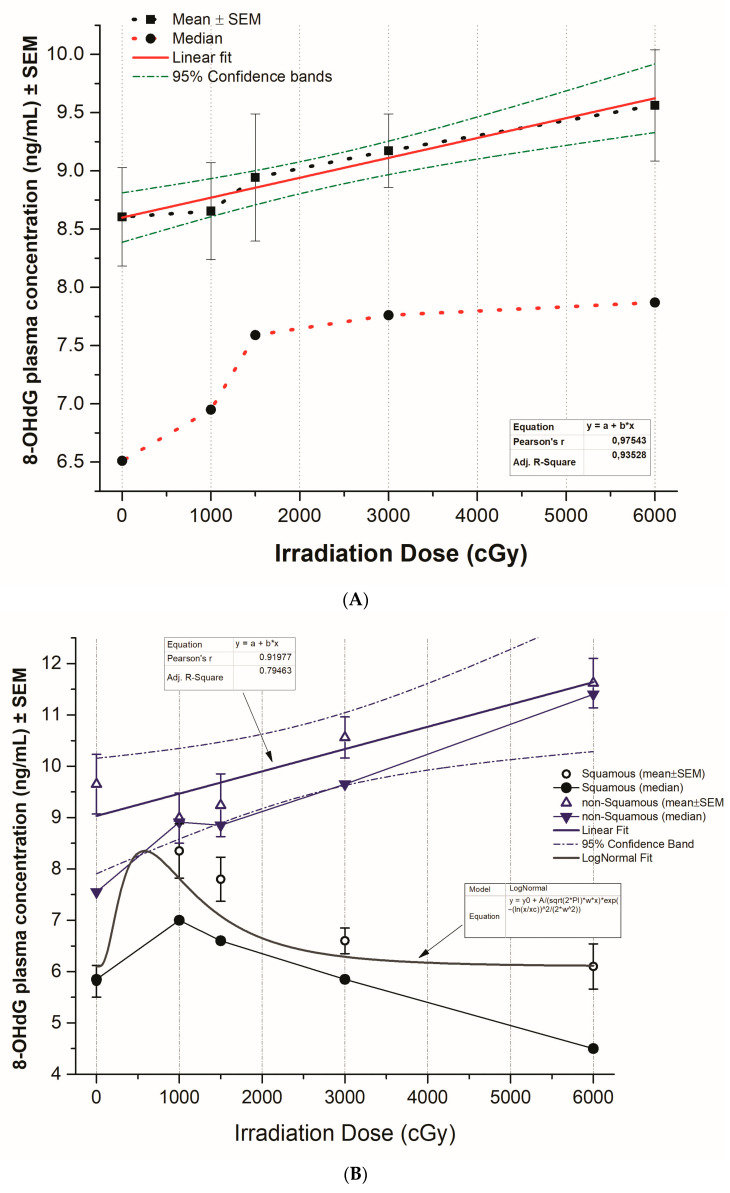

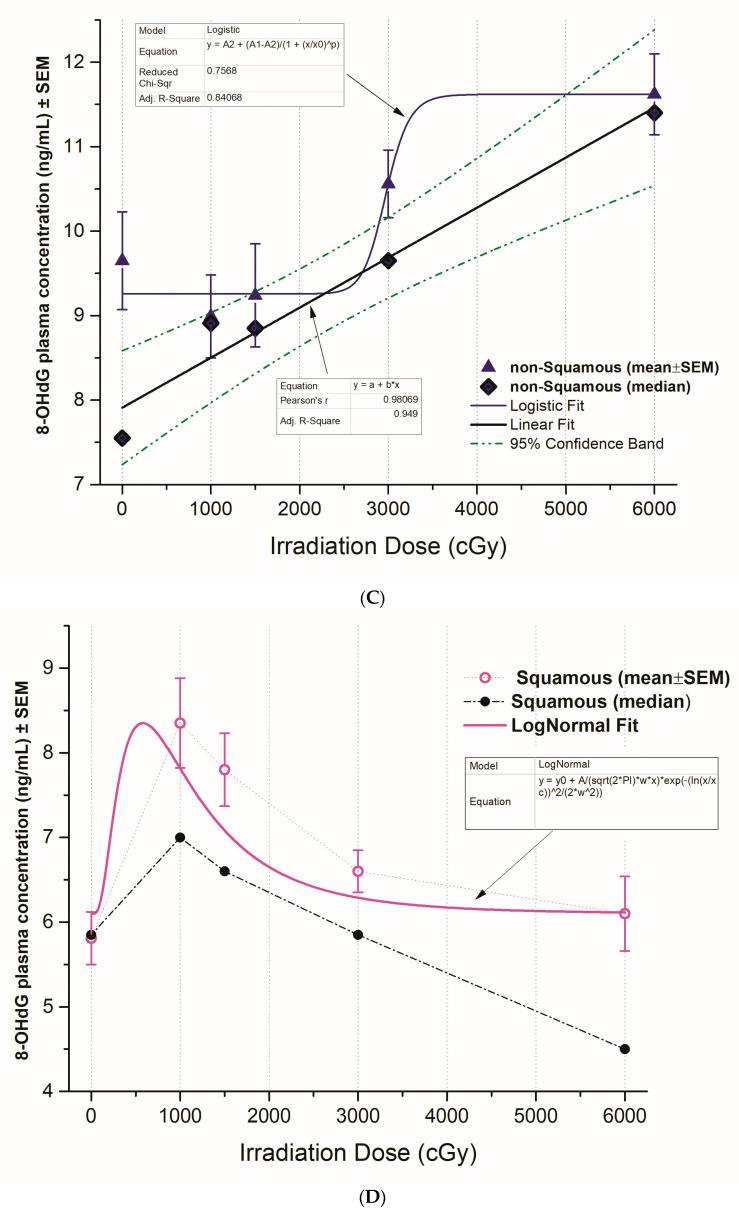
(**A**) Changes in 8-OHdG plasma concentrations (ng/mL) ± SEM in correlation with irradiation dose in all NSCLC patients are presented utilizing linear fit model. (**B**) Correlation curves of 8-OHdG plasma concentration (ng/mL) alterations ± SEM with irradiation dose in NSCLC patients with squamous and non-squamous histology are presented. (**C**) Correlations of 8-OHdG plasma concentration (ng/mL) variations with irradiation dose in NSCLC patients with non-squamous histology are presented utilizing linear and logistic fit models. (**D**) Correlation of 8-OHdG plasma concentration (ng/mL) alterations ± SEM with irradiation dose in NSCLC patients with squamous histology are presented with the use of log normal model.

A significant increase in mean 8-OHdG plasma concentrations in the total of irradiated NSCLC patients was demonstrated (*p* < 0.01). A strong correlation of the 8-OHdG increases with irradiation dose was presented (correlation index r = 0.975; confidence intervals 95%) (Figure 3A).

Moreover, a significant increase in 8-OHdG plasma concentrations in NSCLC with non-squamous histology was demonstrated, especially after 20 Gy doses of irradiation, either with the linear fit model (correlation index r = 0.98 in median and r = 0.92 in average 8-OHdG plasma concentrations) or the logistic fit model (Adj. R-square = 0.84), at confidence intervals (CIs) of 0.95. On the other hand, in patients with squamous histology after an initial significant increase (*p* < 0.01) of 8-OHdG plasma concentrations, especially after 10–15 Gy, a significant decrease (*p* < 0.01) after 20 Gy dosage of irradiation was observed, as it was shown with the LogNormal model (Figure 3B–D).

### 3.4. The Pattern of 8-OHdG Levels in Comparison to Pre-Operative Tumor Volume

**Figure 4:**
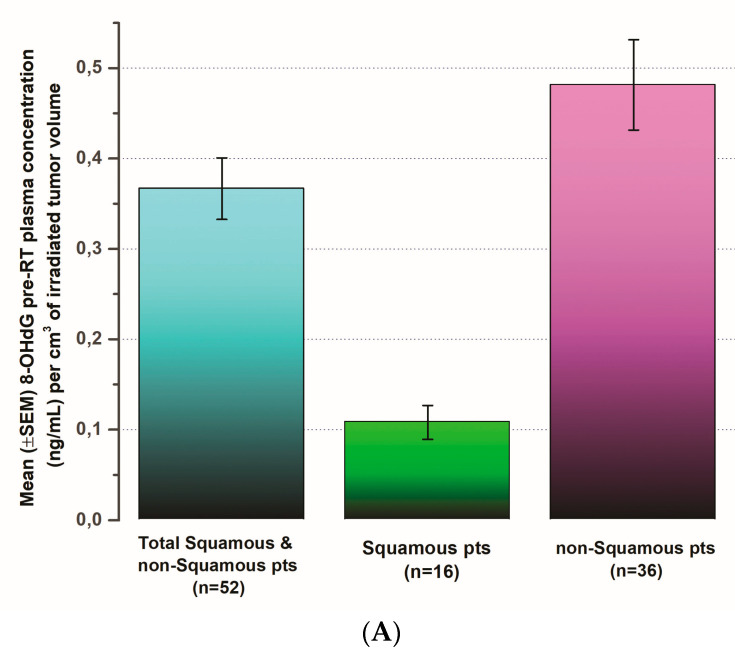

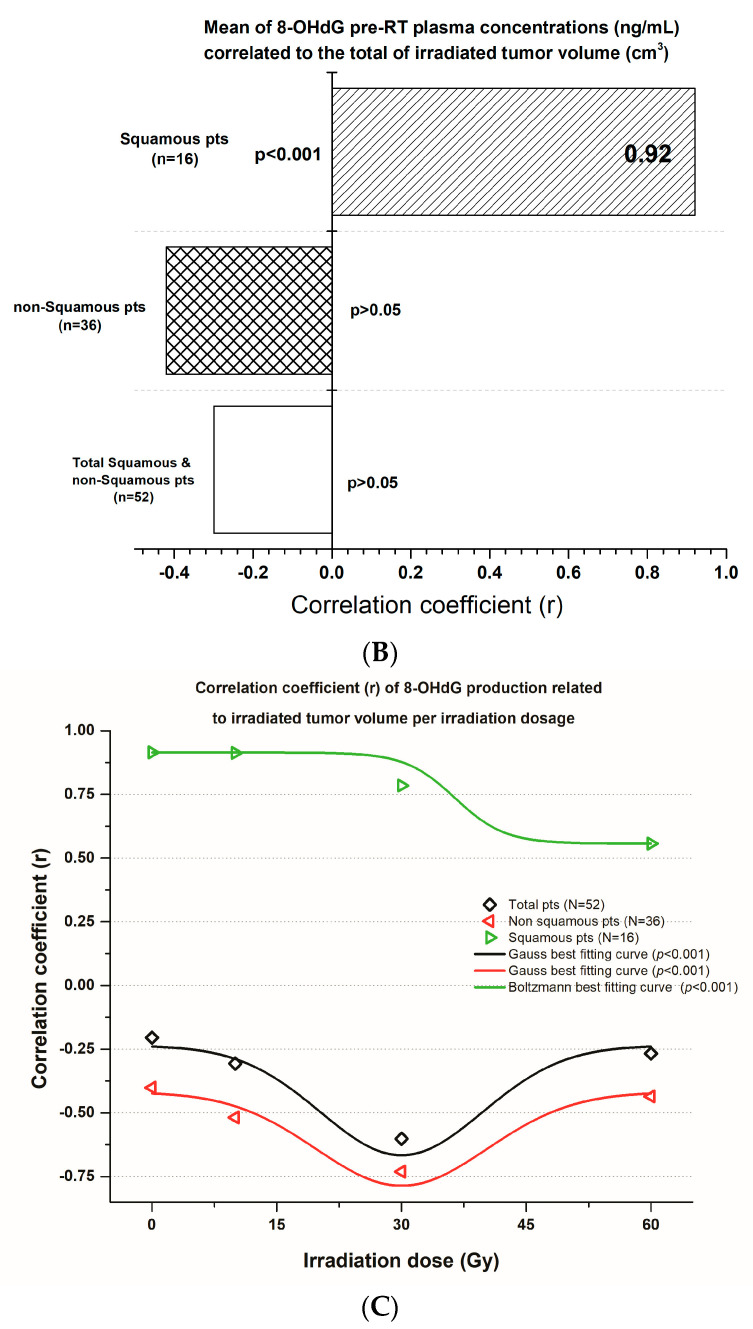
(**A**) The means (±SEM) of 8-OHdG plasma concentrations (ng/mL) per cm^3^ of the irradiated tumor volume before radiation therapy begins at day 0 in total NSCLC patients, as well as in those with squamous and non-squamous histology, are presented. (**B**) Correlations of mean 8-OHdG plasma concentrations (ng/mL) of the total of the irradiated tumor volume (cm^3^) before radiation therapy begins at day 0 in total NSCLC patients, as well as in those with squamous and non-squamous histology, are shown. (**C**) Correlation coefficient (r) of 8-OHdG production in relation to the irradiated tumor volume (cm^3^) per irradiation dosage in total NSCLC patients, as well as in those with squamous and non-squamous histology, are shown. Gauss and Boltzmann best fitting curve models applied.

8-OHdG plasma concentrations (ng/mL) per cm^3^ of the irradiated tumor volume before radiation therapy beginning at day 0 seem to be significantly higher (*p* < 0.001) in NSCLC patients with squamous than those with non-squamous histology (Figure 4A).

Moreover, the mean 8-OHdG plasma concentrations (ng/mL) presented a much stronger correlation (r = 0.92, *p* < 0.001) with the total of the irradiated tumor volume (cm^3^) before radiation therapy started at day 0 in NSCLC patients with squamous histology, while in those with non-squamous histology, no significant correlation was demonstrated (Figure 4B).

On the other hand, the 8-OHdG production in relation to the irradiated tumor volume (cm^3^) per irradiation dosage was strongly correlated in patients with squamous histology at doses of up to 20 Gy (r > 0.90, *p* < 0.001) (Figure 4C). The correlation index (r) reduced at doses higher than 25 Gy (r = 0.72 to 0.52, *p* < 0.001), probably due to the quicker response and shrinkage or necrosis of tumors with squamous histology. In patients with tumors of non-squamous histology, a negative weak correlation between the 8-OHdG production and the irradiated tumor volume (cm^3^) per irradiation dosage appeared. This negative correlation became higher (r = −0.73, *p* < 0.001) at doses of 30 Gy. These results are owed to the higher production of 8-OHdG from tumors with non-squamous histology, before and after irradiation, disproportionately to their size.

### 3.5. 8-OHdG Plasma Concentration Changes and the Tumor Response Rate

**Figure 5:**
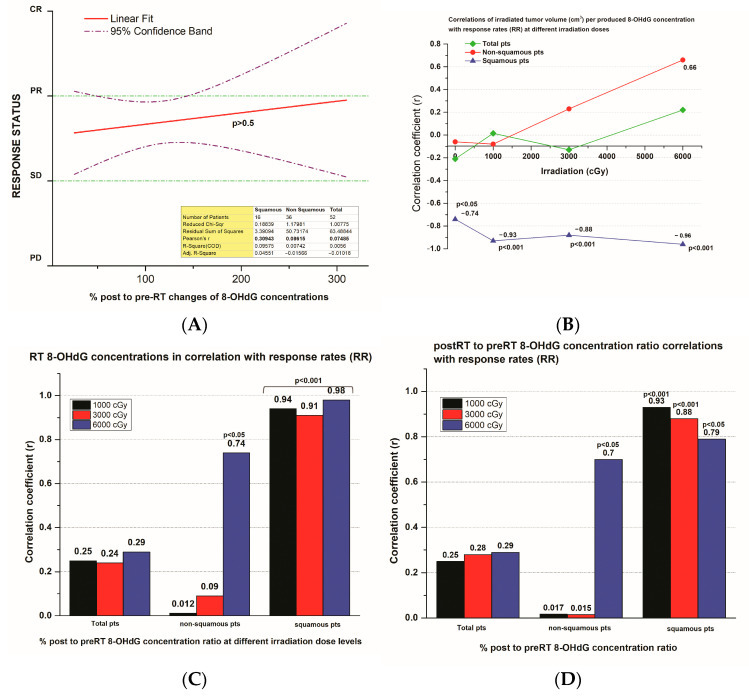

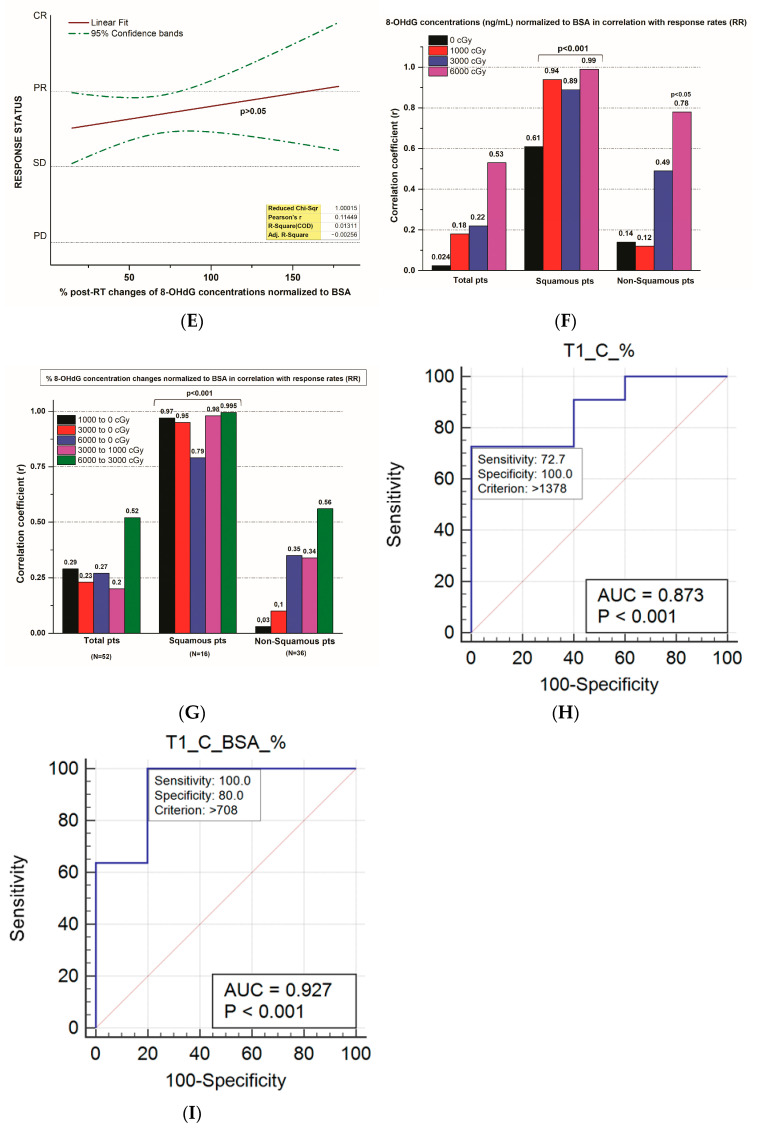
(**A**) A linear fit curve (*p* < 0.05; CIs = 95%) that presents the correlation of the % of post-radiation therapy (post-RT) 8-OHdG plasma concentration changes with the response rate status in the 52 NSCLC patients included in the study. (**B**) The correlations of the irradiated tumor volume (cm^3^) per the respectively produced 8-OHdG plasma concentrations at different irradiation doses with tumor response rates (RR) are shown. (**C**) Histograms that demonstrate the correlation of the 8-OHdG plasma concentration levels with the respective tumor response rates at different irradiation doses in the 52 NSCLC patients and in those bearing tumors with squamous or non-squamous histology. (**D**) Histograms that demonstrate the correlation of the ratios of 8-OHdG plasma concentrations at different irradiation doses to the respective pre-radiation therapy (pre-RT) 8-OHdG plasma concentrations with the corresponding tumor response rates in the 52 NSCLC patients and in those bearing tumors with squamous or non-squamous histology. (**E**) A linear fit curve (*p* < 0.05; CIs = 95%) that presents the correlation of the % of post-radiation therapy (post-RT) 8-OHdG plasma concentration changes normalized to the corresponding BSA, with the response rate status in the 52 NSCLC patients included in the study. (**F**) Histograms that show the correlation of the 8-OHdG plasma concentration levels, normalized with the respective patients’ BSA, and tumor response rates at different irradiation doses in the 52 NSCLC patients and in those bearing tumors with squamous or non-squamous histology. (**G**) Histograms that present the correlation of the ratios of 8-OHdG plasma concentrations at different irradiation doses to the respective pre-radiation therapy (pre-RT) 8-OHdG plasma concentrations, normalized with the respective patients’ BSA, and the corresponding tumor response rates in the 52 NSCLC patients and in those bearing tumors with squamous or non-squamous histology. (**H**) ROC curve of ratio of 8-OHdG plasma concentration at 10 Gy irradiation dose/8-OHdG plasma concentration at 0 Gy irradiation dose (day 0). (**I**). ROC curve of ratio of 8-OHdG plasma concentration at 10 Gy irradiation dose divided by 8-OHdG plasma concentration at 0 Gy irradiation dose (day 0) to BSA.

A very weak positive correlation (correlation index r = 0.07) was presented between the (%) post-radiation therapy (post-RT) 8-OHdG plasma concentration changes and the tumor response rate status in the 52 NSCLC patients included in the study. The correlation was r = 0.09 for patients with tumors of non-squamous and stronger with r = 0.31 for patients of squamous histology, respectively (Figure 5A).

A weak positive correlation (correlation index r = 0.11) between the (%) post-RT 8-OHdG plasma concentration changes normalized to the corresponding BSA and the tumor response rate status in the 52 NSCLC patients also appeared (Figure 5E).

However, the correlations of the irradiated tumor volume (cm^3^) per the respectively produced 8-OHdG plasma concentrations at different irradiation doses with tumor response rates (RR) presented strongly negative correlation (r = −0.93 at 10 Gy dose, *p* < 0.001) in the patients bearing tumors with squamous histology. On the other hand, these correlations were positive (r = 0.66 at 60 Gy dose, *p* < 0.01) in the patients bearing tumors with non-squamous histology. These results concerning these tumors were attributed to the higher production of 8-OHdG, disproportionate to their size (Figure 5B).

A very strong correlation was demonstrated between the 8-OHdG plasma concentration levels or the ratios of 8-OHdG plasma concentrations to the respective pre-radiation therapy (pre-RT) 8-OHdG concentrations and the corresponding tumor response rates at different irradiation doses in patients bearing tumors with squamous histology (r = 0.98 at 60 Gy, r = 0.94 at 10 Gy dose and r = 0.93 at 10 Gy dose, *p* < 0.001; in Figure 5C,D, respectively). On the other hand, a moderate correlation was presented between the 8-OHdG plasma concentration levels or the ratios of 8-OHdG plasma concentrations and the corresponding tumor response rates at different irradiation doses in patients bearing tumors with non-squamous histology (r = 0.74 and r = 0.7 at 60 Gy dose, *p* < 0.05; in Figure 5C,D, respectively).

Similarly, a very strong correlation was demonstrated between the 8-OHdG plasma concentration levels or the ratios of 8-OHdG plasma concentrations to the respective pre-radiation therapy (pre-RT) 8-OHdG concentrations, normalizing with the respective patients’ BSAs, and the corresponding tumor response rates at different irradiation doses in patients bearing tumors with squamous histology (r = 0.99 at 60 Gy, r = 0.94 at 10 Gy dose and r = 0.995 at 60 Gy, r = 0.97 at 10 Gy dose, *p* < 0.001; in Figure 5E,F, respectively).

The response rate of the NSCLC patients with non-squamous histology to irradiation was 58% (21/36 patients presented at least 30% of tumor shrinkage 2 months after radiotherapy). The correlations of the 8-OHdG plasma concentration levels and their changes with the respective tumor response at different irradiation doses were positive (higher at the irradiation dose level of 6000 cGy) but not as significant in comparison to the patients bearing tumors with squamous histology.

A ROC analysis of 8-OHdG plasma concentrations was performed for patients with squamous pathology, which in this series had a response of 68.7% (prevalence of response). When plotting the 8-OHdG plasma concentration at 10 Gy irradiation dose to the 8-OHdG plasma concentration at 0 Gy irradiation dose (at day 0), the AUC was 0.873 (95% CI 0.614–0.984), *p* < 0.0001 and had a Youden index J of 0.727 with an associated criterion value of >1378 as a cutoff, thus producing a sensitivity of 72.7% but a specificity of 100% (Figure 5H). When normalizing this ratio to BSA, the AUC produced was 0.927 (0.684–0.997), *p* < 0.0001, and a Youden index J of 0.800 with an associated criterion value of >708 as a cutoff, thus producing a sensitivity of 100% and a specificity of 80% (Figure 5I).

### 3.6. The Correlation of 8-OHdG Plasma Concentrations and Toxicities

**Figure 6:**
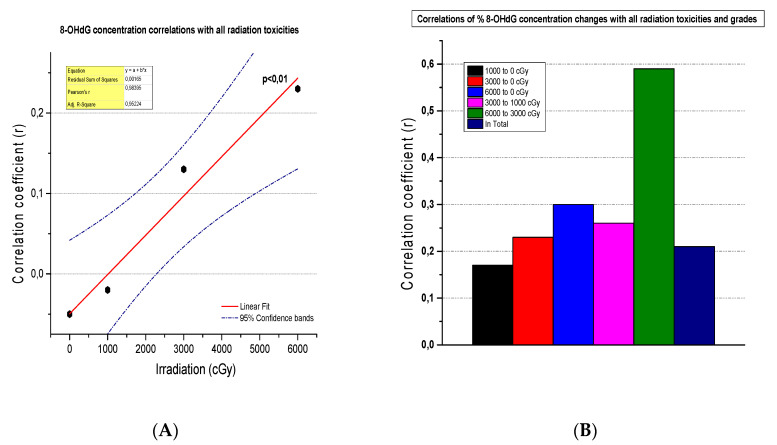
(**A**) A linear fit curve that presents the correlation between the correlation coefficient (r) of 8-OHdG plasma concentrations at different tumor irradiation doses (cGy) and the grade of all toxicities induced by radiation therapy in 52 NSCLC patients. (**B**) Histogram that presents the correlations between the correlation coefficient (r) of % changes in 8-OHdG plasma concentrations at different tumor irradiation dosing levels (cGy) and all toxicities in any grade induced by radiation therapy in 52 NSCLC patients.

A significant and very strong positive correlation presented between the correlation coefficient (r) of 8-OHdG plasma concentrations at different tumor irradiation doses (cGy) and the grade of irradiation-induced toxicities in the 52 NSCLC patients who received radiotherapy (r = 0.98, CIs = 95%, *p* < 0.01) (Figure 6A). On the other hand, weak to moderate but significant (*p* < 0.01) correlations appeared between the correlation coefficient (r) of the % of changes in 8-OHdG plasma concentrations at different tumor irradiation dosing levels (cGy) and all toxicities in any grade induced by radiation therapy in the 52 NSCLC patients. These correlations were significantly stronger (r = 0.59, *p* < 0.001) at 3000–6000 cGy of irradiation dosing levels (Figure 6B).

The most considerable of the common toxicities that induced by radiation therapy in the 52 NSCLC patients were esophagitis, pneumonitis, anemia, fatigue and radiation-induced dermatitis (burn).

**Figure 7:**
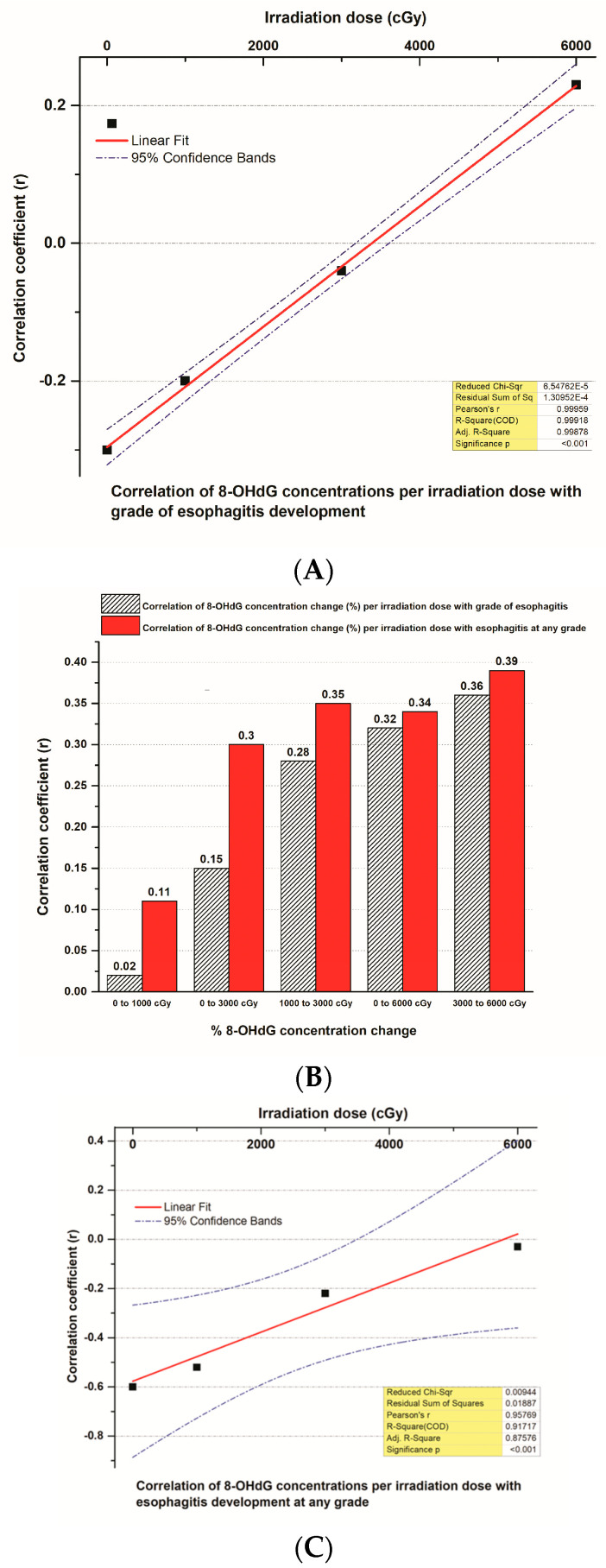
(**A**) A linear fit curve that demonstrates the correlation between the correlation coefficient (r) of 8-OHdG plasma concentrations in different tumor irradiation doses (cGy) and the esophagitis grade produced by radiation therapy in 52 NSCLC patients. (**B**) Histogram that presents the correlations between the correlation coefficient (r) of % changes of 8-OHdG plasma concentrations per different tumor irradiation dosing levels (cGy) and esophagitis grade or at any grade induced by radiation therapy in 52 NSCLC patients. (**C**) A linear fit curve that shows the correlation between the correlation coefficient (r) of 8-OHdG plasma concentrations in different tumor irradiation doses (cGy) and esophagitis at any grade generated by radiation therapy in 52 NSCLC patients.

An impressive significant and very strong positive correlation appeared between the correlation coefficient (r) of 8-OHdG plasma concentrations in different tumor irradiation doses (cGy) and the esophagitis grade generated by the radiation therapy in the 52 NSCLC irradiated patients (r = 0.999, *p* < 0.001, CIs = 95%) (Figure 7A). The correlation of the % changes in 8-OHdG plasma concentrations per different tumor irradiation dosing levels’ (cGy) correlation coefficient (r) with the esophagitis grade or esophagitis at any grade that was induced by radiation therapy and was increased constantly together with the irradiation doses, reaching its higher value at the 3000–6000 cGy dose level (Figure 7B). Moreover, the correlation coefficient (r) of 8-OHdG plasma concentrations in different tumor irradiation doses (cGy) with esophagitis at any grade that was generated by radiation therapy and appeared very strong and significant (r = 0.96, *p* < 0.001, CIs = 95%) (Figure 7C).

**Figure 8:**
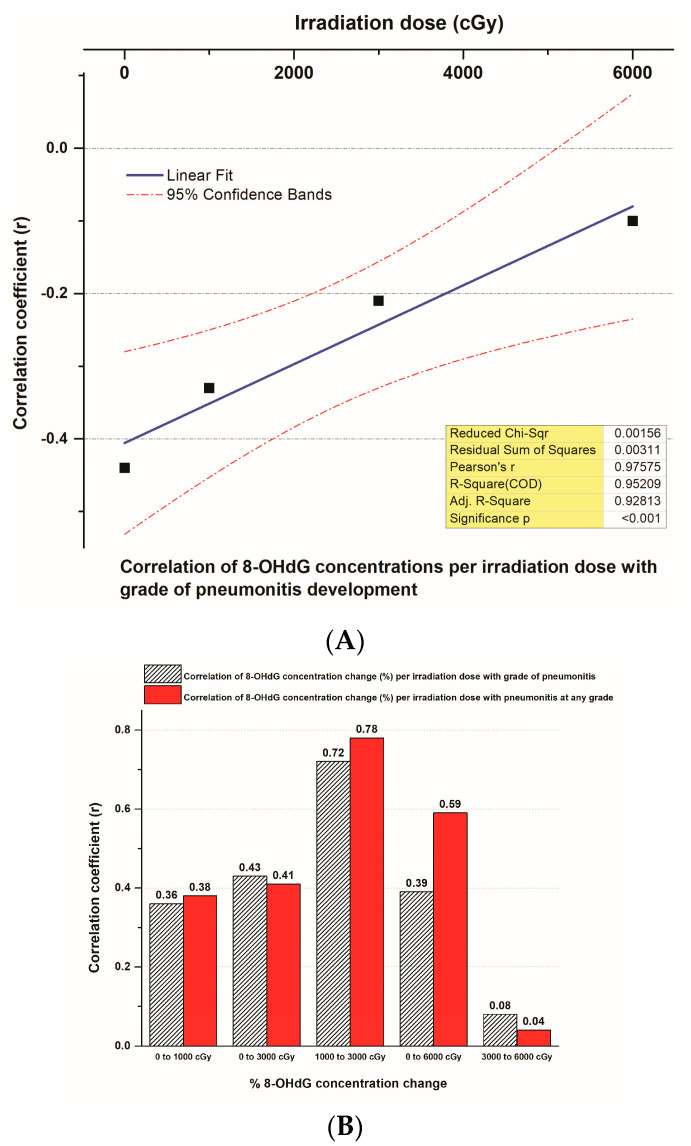

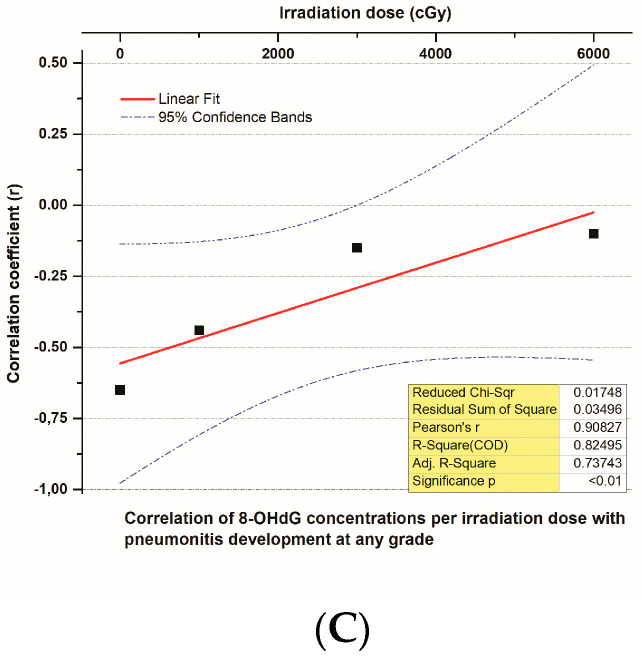
(**A**) A linear fit curve that presents the correlation between the correlation coefficient (r) of 8-OHdG plasma concentrations in different tumor irradiation doses (cGy) and the pneumonitis grade produced by radiation therapy in 52 NSCLC patients. (**B**) Histogram that presents the correlations between the correlation coefficient (r) of % 8-OHdG plasma concentration changes at different tumor irradiation dosing levels (cGy) and pneumonitis grade or at any grade induced by radiation therapy in 52 NSCLC patients. (**C**) A linear fit curve that demonstrates the correlation between the correlation coefficient (r) of 8-OHdG plasma concentrations in different tumor irradiation doses (cGy) and the pneumonitis at any grade induced by radiation therapy in 52 NSCLC patients.

A very strong and significant positive correlation presented between the correlation coefficient (r) of 8-OHdG plasma concentrations in different tumor irradiation doses (cGy) and the pneumonitis grade produced by radiation therapy significance (r = 0.98, *p* < 0.001, CIs = 95%) (Figure 8A). Also, the correlation coefficient (r) of the % of 8-OHdG plasma concentration changes at different tumor irradiation dosing levels and pneumonitis grade or pneumonitis at any grade induced by radiation therapy in the 52 NSCLC patients showed a high and significant positive association at 1000–3000 cGy dosing levels (r = 0.72 and r = 0.78, respectively, *p* < 0.01) (Figure 8B). The correlation between the correlation coefficient (r) of 8-OHdG plasma concentrations in different tumor irradiation doses and the pneumonitis at any grade induced by radiation appears strong (r = 0.91, *p* < 0.01, CIs = 95%) (Figure 8C).

**Figure 9:**
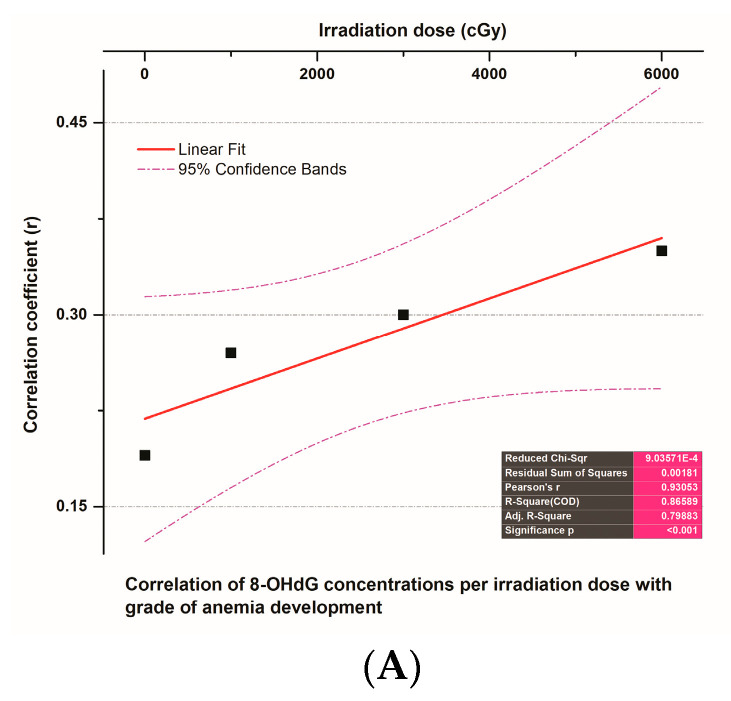

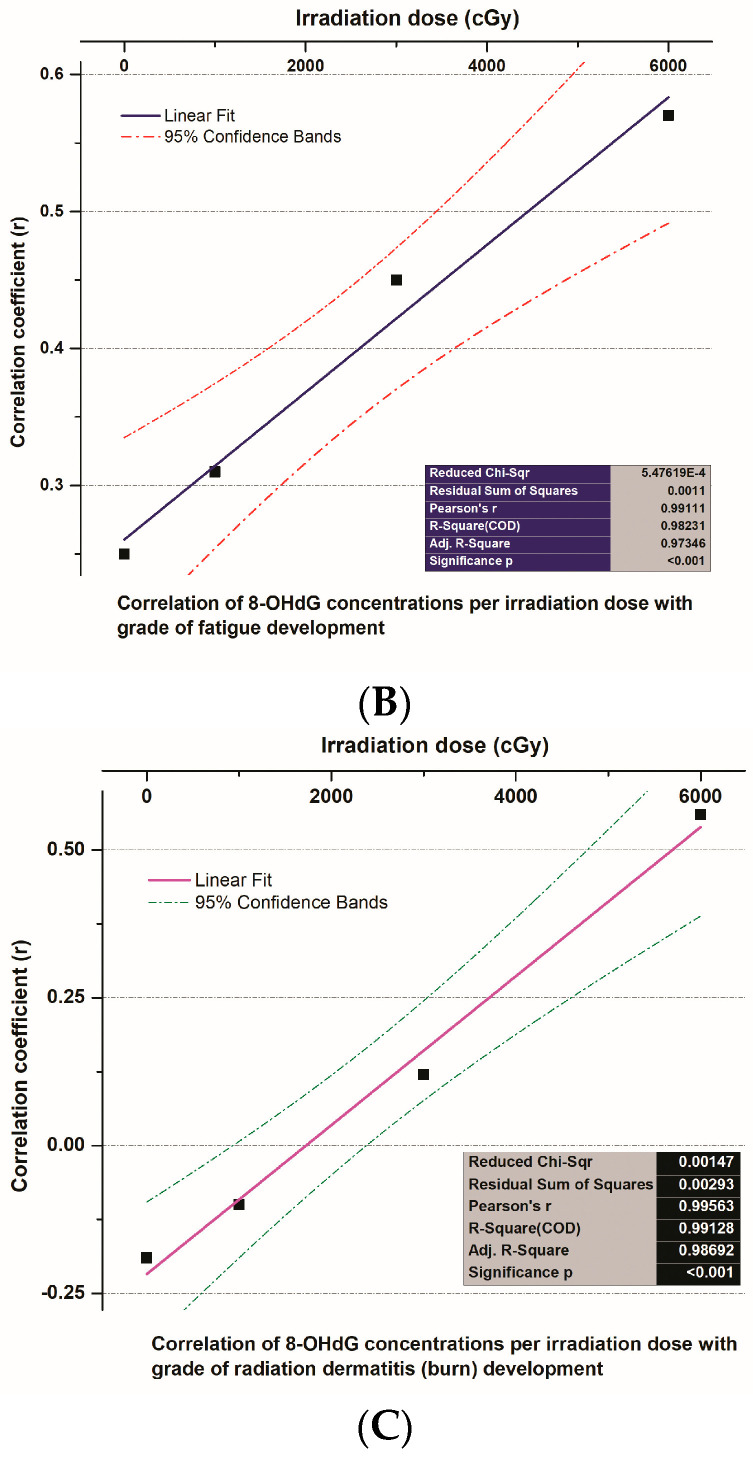
(**A**) A linear fit curve that presents the correlation between the correlation coefficient (r) of 8-OHdG plasma concentrations in different tumor irradiation doses (cGy) and the anemia grade induced by radiation therapy in 52 NSCLC patients. (**B**) A linear fit curve that demonstrates the correlation between the correlation coefficient (r) of 8-OHdG plasma concentrations at different tumor irradiation doses (cGy) and the fatigue grade produced by radiation therapy in 52 NSCLC patients. (**C**) A linear fit curve that shows the correlation between the correlation coefficient (r) of 8-OHdG plasma concentrations in different tumor irradiation doses (cGy) and the dermatitis (burn) grade induced by radiation therapy in 52 NSCLC patients.

Finally, very strong and significant positive correlations were demonstrated between the correlation coefficient (r) of 8-OHdG plasma concentrations in different tumor irradiation doses and grades of anemia (r = 0.93, *p* < 0.001, CIs = 95%) (Figure 9A), fatigue (r = 0.99, *p* < 0.001, CIs = 95%) (Figure 9B) and radiation-induced dermatitis (r = 0.995, *p* < 0.001, CIs = 95%) (Figure 9C) that were developed due to radiation therapy in the 52 NSCLC patients.

Conclusively, the 8-OHdG plasma concentrations that were produced in different tumor irradiation doses closely related to and went with the possibility of the development of radiation-induced toxicities in irradiated NSCLC patients.

## 4. Discussion

In this paper, we tried to elucidate if the blood measurement of 8-OHdG plasma concentrations can serve as a biomarker of DNA damage and explore whether there can be any correlation with the response to radiotherapy treatment in patients with NSCLC. Only stage III patients, with inoperable treatments of lung cancer, that were amenable to and had indications for radical radiotherapy, were included. This provided further homogenicity and comparability of the results and findings. Conclusively, our results showed that measurements of 8-OHdG plasma concentrations, especially with the incorporation of parameters of the patient’s BSA or irradiated tumor volume, could predict safely and with significant accuracy the tumor response rates in NSCLC patients bearing tumors with squamous histology even at initial irradiation doses of 10 Gy.

There have been many studies that report the relation between medically induced irradiation and 8-OHdG measurement either in urine or in blood. Erhola et al. demonstrated elevated 8-OHdG levels in the urine of lung cancer patients after chemotherapy and radiotherapy and the maximal urinary excretion was after cumulative doses of 10 and 30 Gy [18]. Yamazaki et al. suggested that there are different associations between urinary 8-OHdG levels, the type of cancer and when they have their radiation treatment. For example, 8-OHdG levels increased after radiotherapy in prostate cancer, but decreased in patients with cervical cancer or were not significantly different in patients with esophageal or tongue cancer or postoperative breast cancer [19,20].

There has been growing evidence regarding the dose-dependent relationship of 8-OHdG levels and irradiation. Many reports in occupational radiation workers show increased 8-OHdG levels compared to workers not involved with ionizing radiation procedures [12,21,22]. The same has been reported between healthy people not exposed to medical ionizing compared to patients who were previously exposed [18,23]. Gao et al. found that serum 8-OHdG levels decreased after radiotherapy and that there was no linear association between the 8-OHdG levels and the cumulative radiation exposure dose [12]. In our previous work of radiotherapy after breast cancer surgery, blood 8-OHdG levels were significantly increased within the first two weeks of treatment (dose range 10–30 Gy). For the next intervals, where radiation doses were 30–40 Gy 8-OHdG, levels decreased and then remained stable when the radiation dose ranged from 45 to 60 Gy. Thus, there is a threshold over which most cell death has already occurred, thus 8-OHdG levels are decreased. This can be explained by many factors. Firstly, DNA repair systems negate DNA damage so it stops accumulating, and DNA repair takes weeks to months after radiation exposure to take full effect. Secondly, 8-OHdG levels are indirect markers of carcinogenesis, thus they are inherently more increased in cancer patients than in healthy people. Thirdly, besides radiation directly induced oxidative stress, the immune system response causes further generation of ROS [24].

There has been consistent evidence that the serum levels of 8-OHdG in patients exposed to radiotherapy are lower than healthy subjects and there is no relationship between the cumulative/collective radiation dose and the serum levels of 8-OHdG due to the effects of innate DNA repair mechanisms [22]. DNA damage repair is a process that takes time to complete; thus, the adjustments in DNA damage additive output from radiotherapy may be more important in the recovery process (that takes weeks to months) than in the acute initial damage period. Cancer patients have higher 8-OHdG levels than normal healthy individuals and cancer cells once destroyed by radiation therapy result in decreasing serum 8-OHdG levels [12]. In the Gao et al. study, urinary 8-OHdG/creatinine values tend to increase after treatment and return to baseline after two months post-treatment. Patients with resistance to radiation therapy have more pronounced rises in 8-OHdG levels compared to the responders. The decrease in the total mass/volume of the tumor from radiation-induced necrosis is likely to affect these findings; however, the tumor necrosis itself is not responsible for the rise in the 8-OHdG/creatinine ratio [18].

Regarding radiotherapy, 8-OHdG levels and toxicity’s adverse effects, it is not clear if there is a potential correlation. In a study of Crohn’s et al., after a 6-year-follow-up, there seemed to be no significant observation of 8-OHdG levels and adverse effects or long term survival [25]. In this study, we found a significant association between levels of 8-OHdG and the occurrence of esophagitis, pneumonitis, anemia, fatigue and radiation-induced dermatitis.

8-OHdG levels seem to correlate with prognostic factors [26]. Increased urinary 8-OHdG levels are associated with carcinogenesis and prognosis as it has been demonstrated in patients with colorectal cancer and metastatic cancer patients when compared to healthy people or non-metastatic patients suffering from cancer [27]. Furthermore, low 8-OHdG levels are associated with poor prognosis in breast cancer patients [28], whereas increased 8-OHdG levels in serous ovarian carcinoma is associated with lower overall survival and progression-free survival [29]. Also, elevated 8-OHdG levels may be associated with platinum resistance in ovarian cancer, further providing prognostic significance [30]. In our study, there seems to be a prognostic indicator of response in patients with squamous pathology. Specifically, the ROC analysis of 8-OHdG plasma concentrations at 10 Gy irradiation doses to the 8-OHdG plasma concentration at a 0 Gy irradiation dose (at day 0), showed that the AUC was 0.873 (95% CI 0.614–0.984), *p* < 0.0001 and had a Youden index J of 0.727 with an associated criterion value of >1378 as a cutoff, thus producing a sensitivity of 72.7% but a specificity of 100%. When normalizing this ratio to BSA, the AUC produced was 0.927 (0.684–0.997), *p* < 0.0001, and had a Youden index J of 0.800 with an associated criterion value of >708 as a cutoff, thus producing a sensitivity of 100% and a specificity of 80%. This result demonstrates that 8-OHdG plasma concentrations at 10 Gy irradiation are prognostic to the response; however, sensitivity seems to be affected by the confounder of BSA.

In many studies, 8-OHdG levels have been described as non-specific exactly because there seem to be many confounders such as age, sex, smoking, alcohol intake, diet, physical activity and even vitamin status [13], but body mass index (especially in non-smokers) and smoking might be the most important factors affecting urinary 8-OHdG excretion [31]. In our study, all patients upon diagnosis were smokers. However, all of them quit smoking after their diagnosis was made and during the treatment period they continued to abstain from it. Nevertheless, the extent of cellular damage from radiation therapy seems to be the most important factor to increase 8-OHdG levels. Significantly higher levels of 8-OHdG seem to be identified in patients with SCLC receiving radiotherapy compared to NSCLC and to matched controls. However, different pathology or overall volume/burden of disease might contribute [18]. The ratio of urinary 8-OHdG to urinary creatinine was also higher in SCLC than in healthy controls [32]. Furthermore, increasing stage, different pathology and metastasis seem to increase the 8-OHdG levels, probably due to an increased burden of disease [19,27]. In our study, patients were of the same study, and confounders such as renal function, age, sex and smoking were stratified and the pretreatment of ionizing radiation was not permitted. Thus, there seems to be a clear association between 8-OHdG levels and radiotherapy with possible prognostic significance.

It is important to note that the impact of tobacco smoking on overall survival, loco-regional control or overall effectiveness in NSCLC patients undergoing radiotherapy is unclear [33]. There has been conflicting findings in the literature, but smoking may be associated with worse impacts than two-year loco-regional controls for smokers during radiotherapy [34]. Paradoxically, some studies report higher rates of radiation-induced pulmonary toxicity in non-smokers than in smokers [35]

In the literature, multiple efforts to identify predictive biomarkers are identified, such as circulating tumor DNA (ctDNA)-identifying complex SWI/SNF mutations [36]. Genetic analysis confirmed the association between the KEAP1-NRF2 pathway gene alterations and unfavorable survival outcomes, and revealed alterations in FGFR family genes, MET, PTEN, and NOTCH2, as potential novel and independent risk factors of poor post-CRT survival. Patients with EGFR-activating mutations or any oncogenic driver mutations exhibited an improved OS. SNPs in DNA repair-associated XRCC5 (rs3835) and XRCC1 (rs25487) were associated with an increased risk of high-grade esophagitis and pneumonitis, respectively. MTHFR (rs1801133), NQO1 (rs1800566), ZNF217 and POLD1 were risk alleles related to a higher susceptibility to pneumonitis and esophagitis [37].

However, these methods are often complicated and expensive and results are often conflicted by the concurrence of chemotherapy. Our aim was to identify the effects of radiotherapy using oxidative stress with easily reproducible and readily available methods. It is important to also consider the methods used to measure 8-OHdG. In the literature, 8-OHdG has been measured either in urine or in plasma. The most common analytical method used in the analysis of 8-OHdG levels is ELISA, but other methods have been used as well, such as gas chromatography-mass spectrometry analysis (GC-MS) [38], competitive immunochromatography with ICR-001 [23], high-performance liquid chromatography (HPLC) [39] and liquid chromatography-mass spectrometry (LC-MS/MS) [38]. Most studies focused on radiation-related field workers and focused secondarily to patients receiving radiotherapy. Also, the timing of the measurement of samples after radiotherapy varied as well. For example, blood samples were obtained once a week before and after each radiotherapy five times in total [12]. The big difference and hurdle to using urinary 8-OHdG would be the 24-h measurement needed. Although a serum sample may be a more invasive method, it is as accurate and less time consuming for patients.

For the fast detection and quantification of 8-OHdG, ELISA kits have been established, which are both less expensive and time consuming [40]. While ELISA overestimates 8-OHdG levels, it has been shown that there is, regardless, a positive correlation between 8-OHdG and both ELISA or chromatographic methods. Thus, chromatographic methods are preferred when one would want to have a more accurate analysis. However, ELISA on its own seems to be adequate to compare 8-OHdG concentrations [41].

## 5. Conclusions

There seems to be a strong positive relationship between ionizing radiation and 8-OHdG levels in patients with NSCLC. Specifically, patients with squamous pathology seem to have the greatest increase and possible prognostic significance in this subgroup. Biomarkers in cancer treatment are important to predict the failure or success of treatment and it must be personalized. 8-OHdG levels in the serum maybe a useful adjunct in the armamentarium against lung cancer. Further studies are needed to confirm the findings and delineate subpopulations with specific benefits.

## Figures and Tables

**Table 1:** General clinical characteristics of patients with non-small cell lung carcinoma (NSCLC) that included in the study.

Characteristic	Value
Total number (N) of patients	52
Sex (N)	
Male	37
Female	15
Age (y) in average (range)	66.8 (53–76)
Smoking n, (%)	52 (100%)
Histology (N)	
Squamous (SQ)	16
Non-squamous (NSQ)	36
Stage (N)	
IIIA	4
IIIB	17
IIIC	31
Chemotherapy ^1^ (N)	
Before radiotherapy	20
Post radiotherapy	11
Pre- and Post-radiotherapy	14
Concurrently	0
No chemotherapy	7
Overall Response Rate, n (%)	
Squamous	11 (68.8%)
Non-squamous	21 (58.3%)
Body Surface Area (BSA) (m^2^), mean (range)	1.80 (1.45–2.02)
Body Mass Index (BMI) (kg/m^2^), mean (range)	26.76 (20.16–30.42)
Irradiated Tumor Volume (TV) (cm3), mean (range)	
Squamous (SQ)	163.56 (30.5–376.8)
Non-squamous (NSQ)	178.14 (18.1–811.3)

^1^ In all cases, chemotherapy regimen was the combination of carboplatin with docetaxel.

## Data Availability

Data are available upon request to authors D.T.T. and K.O.
